# Mitoautophagy: A Unique Self-Destructive Path Mitochondria of Upper Motor Neurons With TDP-43 Pathology Take, Very Early in ALS

**DOI:** 10.3389/fncel.2019.00489

**Published:** 2019-11-07

**Authors:** Mukesh Gautam, Edward F. Xie, Nuran Kocak, P. Hande Ozdinler

**Affiliations:** ^1^Davee Department of Neurology and Clinical Neurological Sciences, Northwestern University Feinberg School of Medicine, Chicago, IL, United States; ^2^Les Turner ALS Center, Northwestern University Feinberg School of Medicine, Chicago, IL, United States; ^3^Mesulam Cognitive Neurology and Alzheimer’s Disease Center, Northwestern University Feinberg School of Medicine, Chicago, IL, United States; ^4^Robert H. Lurie Comprehensive Cancer Research Center, Northwestern University Feinberg School of Medicine, Chicago, IL, United States

**Keywords:** CSMN, electron microscopy, ALS, mouse models, mitochondria

## Abstract

Mitochondrial dysfunction is one of the converging paths for many neurodegenerative diseases, including amyotrophic lateral sclerosis (ALS), and TDP-43 pathology is the most common proteinopathy detected in ALS and ALS/Frontotemporal lobar degeneration (ALS/FTLD). We recently identified mitochondrial problems in corticospinal motor neurons (CSMN) and in Betz cells of patients with TDP-43 pathology. However, the timing and the extent of mitochondrial defects, and their mode of degeneration have not been revealed. Because it is important to reveal when problems first begin to emerge and whether they are shared or unique, we investigated the health and integrity of mitochondria in CSMN of prpTDP-43^A315T^, PFN1^G118V^, and hSOD1^G93A^ mice at P15 (post-natal day 15)—a very early age in mice without any sign of cellular degeneration.Utilization of immuno-coupled electron microscopy for a detailed surveillance of mitochondria in CSMN and other non-CSMN cells revealed presence of a novel self-destructive path of mitochondrial degeneration, which we named *mitoautophagy*. Mitoauthopgy is different from mitophagy, as it does not require autophagosome-mediated degradation. In contrast, in this novel path, mitochondria can clear themselves independently. We find that even at this early age, all diseased CSMN begin to display mitochondrial defects, whereas mitochondria in non-CSMN cells are healthy. Our findings not only reveal mitoautophagy as a novel path of mitochondrial clearance that occurs prior to neuronal vulnerability, but it also highlights that it is present mainly in the upper motor neurons of prpTDP-43^A315T^ and PFN1^G118V^ mice, which mimic many aspects of the disease in patients with TDP-43 pathology.

## Introduction

Upper motor neuron degeneration is a defining characteristic of numerous neurodegenerative diseases, such as amyotrophic lateral sclerosis (ALS), hereditary spastic paraplegia (HSP), and primary lateral sclerosis (PLS). Therefore, understanding the cellular and molecular mechanisms responsible for their progressive degeneration will have implications in numerous diseases in which voluntary movement is affected.

Motor neuron circuitry is one of the most complex circuitries, responsible for the initiation and modulation of voluntary movement. Upper motor neurons—the corticospinal motor neurons (CSMN) in mice and the Betz cells in humans—are the key cortical component of this circuitry. They are unique in their ability to collect and integrate thousands of different types of cortical input and to translate this into a message that is transmitted to the spinal cord targets for the voluntary movement to be initiated. Building evidence reveals that upper motor neuron degeneration is an early event in ALS, and that the cortical component of the disease raise the “red flag” in patients much earlier than symptom onset, with increased hyper excitation followed by hypo excitation (Geevasinga et al., 2016; Van den Bos et al., 2018), and disintegration of their apical dendrite with spine loss (Genc et al., 2017).

Mutations in the *SOD1* gene were the first most significant discovery in the field of ALS. SOD1 mouse models also mimicked many aspects of disease pathology observed in patients and they are widely used to reveal the underlying mechanisms that lead to motor neuron vulnerability and degeneration. Even though absence of SOD1 does not lead to degeneration, mutant SOD1 has gain of toxic function, which contributes to oxidative and ER stress, axon transport defects, and mitochondrial problems, among many others.

Since the discovery of SOD1 mutations, many more genes are identified to be either directly linked or associated with ALS, and *Profilin 1* (*PFN1*) gene is one of them. Profilin plays an important role in cytoskeleton maintenance as it binds to actin and regulates assembly and disassembly of microtubules (Nejedla et al., 2016). Mutations in *PFN1* gene have been detected in ALS patients and mouse models of Profilin have been generated in an effort to reveal the underlying mechanisms that lead to motor neuron degeneration (Yang et al., 2016; Fil et al., 2017).

Mutations in the *TARDBP* gene were also detected in a broad spectrum of ALS patients. TDP-43, which is encoded by the *TARDBP* gene, is a DNA/RNA binding protein that is implicated in many cellular functions, most important of those is the RNA metabolism (Cohen et al., 2011). TDP-43 regulates various aspect of RNA metabolism including RNA processing, miRNA biogenesis, and RNA splicing (Ling et al., 2013). TDP-43 is found to be associated with mitochondria and mitochondrial defects with respect to TDP-43 pathology are also beginning to emerge (Davis et al., 2018). Among many of the mouse models generated to investigate TDP-43 pathology, the prpTDP-43^A315T^ mouse offers a good tool, as the model mimics many aspects of the disease (Wegorzewska et al., 2009) and displays progressive CSMN loss (Gautam et al., 2019).

TDP-43 pathology is defined as the aggregation of proteins that include phosphorylated form of TDP-43 protein and it is one of the most commonly observed proteinopathies in the cortex of ALS and ALS/FTLD patients (Coan and Mitchell, 2015; Cykowski et al., 2017; Shenouda et al., 2018). It is important to note that even in the absence of mutations in *TARDBP* gene, the TDP-43 pathology is observed in the brains of patients, including the ones that had mutations in their *PFN1* gene (Wu et al., 2012; Smith et al., 2015). Interestingly, patients with *SOD1* mutations were mostly devoid of TDP-43 pathology (Mackenzie et al., 2007; Robertson et al., 2007).

The well-characterized mouse models offer many advantages to reveal the cellular defects that occur in motor neurons that become vulnerable to disease. Emerging evidence also demonstrates that when Betz cells in patients and CSMN in mice are compared at a cellular level, the findings are comparable and that upper motor neurons in mice and humans become vulnerable due to similar causes (Genc et al., 2017). For example, within the context of TDP-43 pathology, both CSMN and Betz cells display nuclear membrane, ER and mitochondrial defects (Gautam et al., 2019).

Similar to TDP-43 pathology, mitochondrial defects emerge as one of the converging problems that occur broadly in many neurodegenerative diseases. Mitochondria display ultrastructural defects in the upper motor neurons of ALS patients and mouse models of ALS (Gautam et al., 2016, 2019). However, it is important to know if these defects are restricted to upper motor neurons in the motor cortex, if the type and the extent of mitochondrial defects are comparable among different underlying mechanisms, and how early they are initiated. P15 (post-natal day 15) is a time when new-born pups are still in the nest and this age may correspond to childhood when no behavioral abnormalities are detected. We thus selected P15 as the time of investigation to assess any early mitochondrial damage which occurs in CSMN that become diseased due to TDP-43 pathology, mSOD1 toxicity and lack of profilin function, three independent but clinically relevant causes of ALS in patients.

Our findings, which utilize immuno-coupled electron microscopy, revealed the presence of a novel self-destructive path mitochondria take to eliminate itself. Since this pathway of mitochondrial degradation was very distinct and different from mitophagy, we coined the term “mitoautophagy” to emphasize the fact that mitochondria are capable of self-destruction without the need to be engulfed by lysosomes or autophagasomes. Interestingly, not all diseased CSMN utilized mitoautophagy to the same extent; it was prominent mainly in the CSMN of prpTDP-43^A315T^ and PFN1^G118V^, but not in hSOD1^G93A^ mice. None of the non-CSMN cells contained defective mitochondria at this age, suggesting that defects are more restricted to CSMN. Here, we not only identify mitoautophagy as a novel path for mitochondrial degeneration, but also reveal that mitochondrial defects occur very early in upper motor neurons and that there are distinct differences in the mode of mitochondrial degeneration. The mitochondrial abnormalities detected during post-natal development could indeed be one of the first defects that lead to upper motor neuron vulnerability especially within the context of TDP-43 pathology.

## Materials and Methods

### Mice

All animal experiments were performed in compliance with the standards set by National Institutes of Health and were approved by the Northwestern University Animal Care and Use committee. The following mouse strains were used in this study: WT (wild-type); prpTDP-43^A315T^ mice (Jackson Laboratory, stock#. 010700), which uses prion (prp) promoter to drive the mutant form of hTDP-43; hSOD1^G93A^ (B6SJL-Tg(SOD1*G93A)1Gur/J; The Jackson Laboratory, stock# 002726), which overexpresses the mutant form of hSOD1 and have been mated to the BL6 background for more than seven generations prior to inclusion in this study; and PFN1^G118V^ (gift by Dr. Kiaei, University of Arkansas, USA), which overexpress the mutant form of human PFN1. All mice used in this study were in the BL6 background and the WT littermates were comparable.

### Tissue Collection and Processing

Mice were deeply anesthetized with intraperitoneal injection of Ketamine (90 mg/kg) and Xylazine (10 mg/kg; Fort Dodge Animal Health, Fort Dodge, IA, USA) prior to transcardial perfusion with 0.12 M phosphate buffered (PB) containing 2% paraformaldehyde (PFA) and 0.5% glutaraldehyde. Intact cortices were dissected out, post-fixed in 2% PFA for 1 h at room temperature and stored long-term for later use in phosphate buffered saline (PBS) with 0.01% sodium azide at 4°C.

### Immunohistochemistry

Brains were post-fixed in 4% PFA overnight and sectioned at 50 μm (Leica VT1000S, Leica Inc., Nussloch, Germany). Floating sections were incubated in blocking solution (PBS, 0.05% bovine serum albumin, 2% fetal bovine serum, 1% Triton X-100 and 0.1% saponin) for 30 min prior to addition of primary antibodies: rabbit anti-LC3B (1:500, Invitrogen, Carlsbad, CA, USA), mouse anti-ATP5A (1:250, Abcam, Cambridge, MA, USA). The sections were incubated in primary antibody overnight at 4°C. After extensive washes, sections were incubated in appropriate secondary antibodies in blocking solution: Alexa Fluor anti-mouse 488 (1:1,000, Invitrogen, Carlsbad, CA, USA), and Alexa Fluor anti-rabbit 555 (1:1,000, Molecular Probes, Eugene, OR, USA) for 2 h at room temperature. The sections were counterstained with DAPI and imaged using Nikon A1R (A) spectral confocal microscope.

### Immunohistochemistry Coupled With EM

Brain were coronally sectioned at 50 μm (Leica VT1000S, Leica Inc., Nussloch, Germany). The sections were post-fixed in 2% PFA and 0.5% glutaraldehyde for 1 h at room temperature, they were cryoprotected with glycerol–dimethylsulfoxide (DMSO) mixture followed by freeze–thaw at least four times and treated with 1% sodium borohydrate. They were then treated with 0.3% H_2_O_2_–10% methanol in TBS (100 mm Tris–HCl and 150 mM NaCl, pH 7.6) and 5% normal goat serum–1% bovine serum albumin in TBS to block non-specific binding of primary antibody. They were incubated overnight with rabbit anti-Ctip2 antibody (1:500, Sigma Aldrich, St. Louise, MO, USA). Biotinylated goat anti-rabbit IgG (1:500, Vector Laboratories, Burlingame, CA, USA) were used as a secondary antibody, and diaminobenzidine (DAB) was applied as the chromogen (ABC Elite Kit, Vector Laboratories, Burlingame, CA, USA). The sections were then post-fixed in buffered 2% osmium tetroxide (OsO_4_, Electron Microscopy Sciences, Hatfield, PA, USA), rinsed with distilled water and stained in 1% uranyl acetate (Electron Microscopy Sciences, Hatfield, PA, USA), again rinsed with distilled water, dehydrated in ascending grades of ethanol with transition fluid propylene oxide (Electron Microscopy Sciences, Hatfield, PA, USA) and embedded in resin mixture with Embed 812 (Electron Microscopy Sciences, Hatfield, PA, USA) and cured in a 60°C oven for 3 days. The sections in which primary motor cortex was present and visible under bright field illumination on a dissecting scope were selected. Approximately, 5 mm wide × 7 mm long piece of the motor cortex from these sections was dissected under the microscope, mounted on resin block and was sectioned on a Leica Ultracut UC6 ultramicrotome (Leica Inc., Nussloch, Germany). Seventy nanometers thin sections were collected on 200 mesh copper–palladium grids. Grids were counter stained with 8% radioactive depleted uranyl acetate and 0.2% lead citrate. Grids were examined on FEI Tecnai Spirit G2 TEM (FEI company, Hillsboro, OR, USA), and digital images were captured on a FEI Eagle camera.

### Analyses of Mitochondria

High magnification images of CSMN were taken using electron microscope. CSMN were identified by their large size, and presence of a large nucleus that is labeled with high-levels of Ctip2 expression (Arlotta et al., 2005). Mitochondria in CSMN were counted by using either analyses toolbox of Photoshop (Adobe Inc., San Jose, CA, USA) or ImageJ (NIH) software. Counting was performed by at least two independent people blinded to the identity and genotype of the samples.

### Statistical Analyses

Prism software (GraphPad Software Inc., La Jolla, CA, USA) was used for all statistical analyses. For each quantification at least *n* = 3 mice were used for each genotype and group. Statistical analyses were based on the average numbers for each mouse, and not based on total individual number of cells or organelle. When counting organelles present inside the cell, sampling was not applied. All organelle that are present in the image were counted without bias or elimination. About 20 different cells were analyzed per mouse per genotype and 20–30 mitochondria were present and counted in each cell. Even though the total number of mitochondria differs from cell to cell, the percentage of defective mitochondria was quantified per cell and the averages of cells determined the quantitative measure for each mouse, which are presented in the bar graphs. The total number of mitochondria counted and the total number of cells analyzed for each quantitative measure are presented in Supplementary Table S1. D’Agostino and Pearson normality test was performed on all data sets. Either Student’s *t*-test or one-way analysis of variance (ANOVA) with *post hoc* Dunn’s multiple comparison test was used to determine statistical differences between experimental groups depending on the genotype and the disease group. Data are shown as mean ± SEM of at least three replicates and is representative of three independent experiments unless otherwise stated, and statistically significant differences were taken at *p* < 0.05, and *p*-values or adjusted *p*-values are reported in the text.

## Results

### Mitochondrial Defects Are Observed Selectively in Diseased CSMN

**Figure 1:**
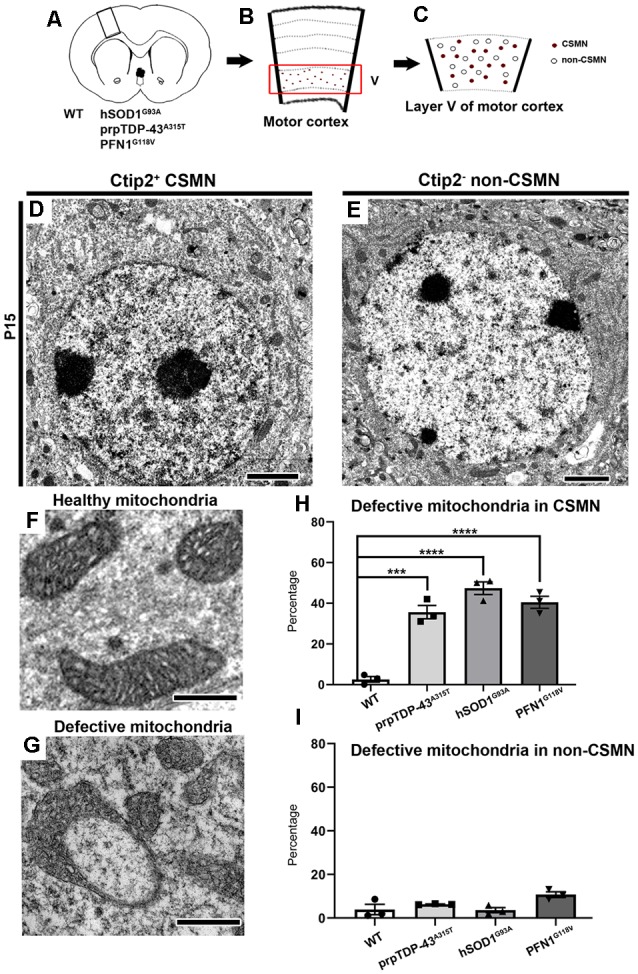
Corticospinal motor neurons (CSMN) display mitochondrial defects at P15. **(A)** Schematic representation of coronal section of brain isolated from wild type (WT), prpTDP-43^A315T^, hSOD1^G93A^ and PFN1^G118V^ mice at P15. **(B)** A representative drawing of motor column showing layer V of motor cortex in the box. **(C)** A representative sketch of layer V of motor cortex depicting CSMN (colored dots) and non-CSMN cells (white circles). **(D)** A representative electron micrograph of Ctip2+ CSMN. **(E)** A representative electron micropgraph of Ctip2- non-CSMN cell. **(F)** A representative image of healthy mitochondria. **(G)** A representative image of defective mitochondria. **(H)** Bar graph representation of percentage of defective mitochondria in the CSMN of WT, prpTDP-43^A315T^, hSOD1^G93A^ and PFN1^G118V^ mice **(I)** Bar graph representation of percentage of defective mitochondria in non-CSMN cells of WT, prpTDP-43^A315T^, hSOD1^G93A^ and PFN1^G118V^ mice. *p*-value *** = 0.0001, **** <0.0001 Scale bar: **(D,E)** = 2 μm **(H,G)** = 500 nm.

In an effort to visualize the cytoplasm of diseased CSMN and to investigate whether any of the organelles display defects very early in the disease, immuno-coupled EM is performed on prpTDP-43^A315T^, hSOD1^G93A^, and PFN1^G118V^ mice at P15 (Figure 1A). These mouse models were chosen because progressive CSMN degeneration was previously determined with detailed cellular analysis, recapitulating human pathology (Ozdinler et al., 2011; Fil et al., 2017; Gautam et al., 2019). WT mice are used as healthy controls and they are obtained from the littermates of the diseased mice. At P15, the pups are in the nest, and at this very early age, none of the disease models display any behavioral or cellular defects. P15 is chosen for the time of investigation because any intracellular defect detected at this age would potentially precede and contribute to neuronal vulnerability.

CSMN were marked by their large, pyramidal soma located in the layer V of the motor cortex and presence of prominent Ctip2 expression in their large nucleus (Figures 1B–D). CSMN were surrounded by many other non-CSMN cells that lack Ctip2 expression (Figure 1E), and it was thus possible to distinguish CSMN from non-CSMN cells on the same planar section (Figures 1D,E). The layer V of the motor cortex was isolated, trimmed and prepared for electron microscopy (EM) imaging (Figures 1B,C). Initial investigation of the potential intracellular defects revealed the presence of numerous mitochondria that were defective, mainly in CSMN. Mitochondria were considered defective if their shape was extraordinarily distorted, inner membrane compromised, they formed a ring-like structure or if their outer membrane was broken. In normal healthy controls and in cells that are not CSMN, the mitochondria appeared normal with proper structural integrity (Figure 1F). On the other hand, mitochondria of CSMN in prpTDP-43^A315T^, hSOD1^G93A^ and PFN1^G118V^ mice displayed numerous ultrastructural defects (Figures 1G,H; percentage of defective mitochondria in CSMN: WT: 2.57 ± 1.4%; prpTDP-43^A315T^: 35.63 ± 3.3%, adjusted *p*-value = 0.0001, hSOD1^G93A^: 47.41 ± 3.1% adjusted *p*-value = 0.0001, PFN1^G118V^: 40.47 ± 2.98%, adjusted *p-value* = 0.0001). In contrast, mitochondria of non-CSMN cells were healthy (Figure 1I; percentage of defective mitochondria in non-CSMN: WT: 3.92 ± 1.88%; prpTDP-43^A315T^: 6.21 ± 1.46%, adjusted *p*-value = 0.675; hSOD1^G93A^: 3.27 ± 1.44%, adjusted *p-value* = 0.998; PFN1^G118V^: 9.23 ± 2%, adjusted *p*-value = 0.064).

### Defective Mitochondria Communicate With Each Other and Form Connections With Autophagosomes

**Figure 2:**
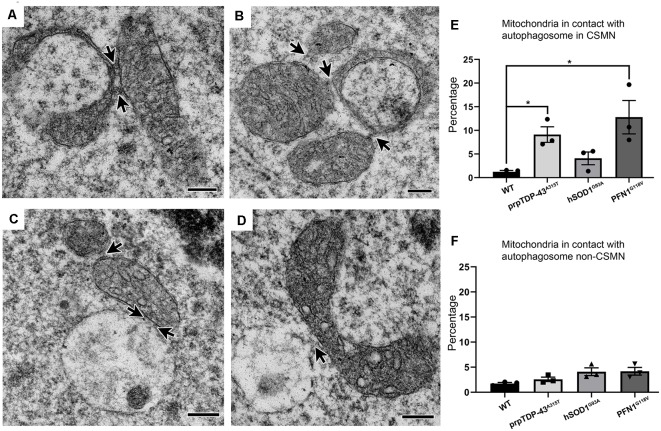
Defective mitochondria in diseased CSMN cross-talk with each other and some are attached to autophagosomes. **(A)** Representative electron micrograph image of defective mitochondria in an act of cross-talk with other mitochondria (arrows). **(B)** Representative image of Stage II mitochondria communicating with two different mitochondria (arrows). **(C,D)** Representative electron micrograph of defective mitochondria attached with an autophagosome. **(E)** Quantification of percent distribution of mitochondria in contact with an autophagosome in CSMN of WT, prpTDP-43^A315T^, hSOD1^G93A^ and PFN1^G118V^ mice. **(F)** Quantification of percent distribution of mitochondria in contact with an autophagosome in non-CSMN cell of WT, prpTDP-43^A315T^, hSOD1^G93A^ and PFN1^G118V^ mice. *p*-value * <0.01. Scale bar: **(A–D)** = 200 nm.

Previous work from many other groups already identified the detailed steps that lead to the autophagasome formation and systemic elimination of mitochondria in cells (Evans and Holzbaur, 2019). Similar to these observations, the high magnification images revealed the presence of a “linkage” or tethering among mitochondria, forming direct connections with each other (Figures 2A,B, arrows). In some instances, mitochondria with different sizes and stages of degeneration were grouped (Figure 2B). Some mitochondria were also found either in close proximity to or in direct contact with autophagosomes (Figures 2C,D). About 10% of mitochondria were associated with autophagosomes in CSMN of prpTDP-43^A315T^ (9.1 ± 2%, adjusted *p*-value = 0.009), and PFN1^G118V^ (12.79 ± 4% adjusted *p*-value = 0.03) mice, whereas CSMN of hSOD1^G93A^ was comparable to that of WT (WT: 1.23 ± 1%; hSOD1^G93A^: 4.08 ± 2% adjusted *p*-value = 0.107, Figure 2E). Mitochondria of non-CSMN neurons were not in contact with autophagosomes (WT: 1.73 ± 1%; prpTDP-43^A315T^: 2.57 ± 1%, adjusted *p*-value = 0.354; hSOD1^G93A^: 4.09 ± 1%, adjusted *p*-value = 0.323 and PFN1^G118V^: 4.17 ± 1%, adjusted *p-value* = 0.160; Figure 2F).

### Diseased Mitochondria Self-Destruct *via* Mitoautophagy

**Figure 3:**
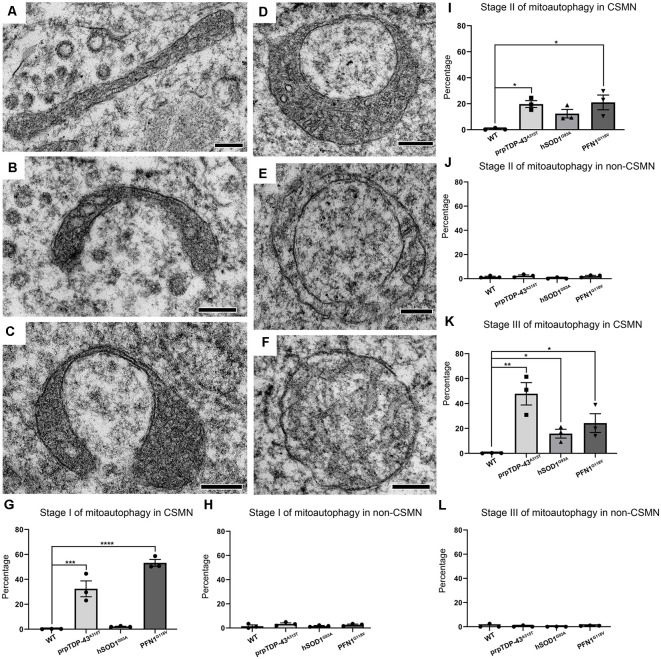
Diseased CSMN display different stages of mitoautophagy. **(A–C)** Representative examples of Stage I of mitoautophagy. **(D,E)** Representative examples of Stage II of mitoautophagy. **(F)** Representative examples of Stage III of mitoautophagy. **(G)** Quantification of percent mitochondria detected in Stage I of mitoautophagy in CSMN. **(H)** Quantification of percent mitochondria detected in Stage I of mitoautophagy in non-CSMN cells. **(I)** Quantification of percent mitochondria detected in Stage II of mitoautophagy in CSMN. **(J)** Quantification of percent mitochondria detected in Stage II of mitoautophagy in non-CSMN cells. **(K)** Quantification of percent mitochondria detected in Stage III of mitoautophagy in CSMN. **(L)** Quantification of percent mitochondria detected in Stage III of mitoautophagy in non-CSMN cells. *p*-value * = 0.012, ** = 0.0012, *** = 0.0008, **** <0.0001. Scale bar: **A–F** = 200 nm.

Close examination of the cytoplasmic content of CSMN in WT, hSOD1^G93A^, prp-TDP-43^A315T^, and PFN1^G118V^ mice revealed the presence of numerous mitochondria with different patterns and stages of degeneration (Figure 3). In an effort to perform a more systemic surveillance, and to reveal presence of potential modes or stages of degeneration, more than 200 cells were analyzed (Supplementary Table S1). Some mitochondria were found to be stretched and elongated (Figure 3A) while others were forming a U-shape (Figures 3B,C). Interestingly, there were numerous examples of mitochondria that had both ends connected, developing a ring-like structure (Figure 3D). Since EM images are a snapshot in time and many mitochondria could be caught at different stages, we investigated whether these distinct forms and shapes of mitochondria could represent a consequential event that takes place in the neuron. Upon analyses of 200 neurons from different genotypes (Supplementary Table S1), we began to realize the presence of a line of events that led to mitochondrial degeneration in a very unique way. We named this phenomena *mitoautophagy* to emphasize the fact that it is a self-destructive path for mitochondrial degeneration.

*Mitoautophag*y started first with elongation, stretching and folding of mitochondria (Stage I; Figures 3A–C), followed by unification of folded ends (Stage II; Figure 3D), and leading to disintegration of first the inner membrane followed by the outer membrane (Stage III; Figures 3E,F). Since CSMN that become diseased due to different underlying causes may undergo this novel mitoautophagy at different rates and to a different extent, the percentage of mitochondria that are at different stages of mitoautophagy in CSMN of WT, hSOD1^G93A^, prpTDP-43^A315T^, and PFN1^G118V^ mice are quantified. Numerous mitochondria in diseased CSMN were in either Stage I (WT: 0.18 ± 1%; prpTDP-43^A315T^: 32.43 ± 6.36%, adjusted *p*-value = 0.0008, hSOD1^G93A^: 2.14 ± 0.24%, adjusted *p*-value = 0.976, PFN1^G118V^: 53.24 ± 2.71%, adjusted *p*-value = 0.0001 Figure 3G), Stage II (WT: 0.59 ± 0.43%; prpTDP-43^A315T^: 19.37 ± 2.65%, adjusted *p*-value = 0.02, hSOD1^G93A^: 12.38 ± 3.26%, adjusted *p*-value = 0.0001, PFN1^G118V^: 21.01 ± 5.66%, adjusted *p*-value = 0.014; Figure 3I) or Stage III (WT: 0.21 ± 0.1%; prpTDP-43^A315T^: 47.8 ± 8.97%, adjusted *p*-value = 0.002, hSOD1^G93A^: 15.83 ± 3.51%, adjusted *p*-value = 0.011, PFN1^G118V^: 24.32 ± 7.52%, adjusted *p*-value = 0.032; Figure 3K). These results suggest that mitoautophagy occurred not only in the CSMN of prp-TDP-43^A315T^ mice, but was also present in CSMN of PFN1^G118V^ mice, albeit at different rates. Mitochondria in CSMN of hSOD1^G93A^ mice displayed low levels of mitoautophagy.

Interestingly, non-CSMN cells that are located in close proximity to CSMN in layer V of the motor cortex contained healthy mitochondria and were comparable among all genotypes and stages (Stage I: WT: 1.49 ± 1%; prpTDP-43^A315T^: 3.38 ± 1%, adjusted *p*-value = 0.607; hSOD1^G93A^: 1.48 ± 1%, adjusted *p*-value = 0.999 and PFN1^G118V^: 2.48 ± 1%, adjusted *p-value* = 0.902, Figure 3H), Stage II (WT: 1.57 ± 1%; prpTDP-43^A315T^: 2.43 ± 1% adjusted *p*-value = 0.937; hSOD1^G93A^: 0.573 ± 1%, adjusted *p*-value = 0.879 and PFN1^G118V^: 2.81 ± 1.2%, adjusted *p*-value = 0.814, Figure 3J) and Stage III (WT: 0.85 ± 1%; prpTDP-43^A315T^: 0.45 ± 0.39% adjusted *p*-value = 0.967; hSOD1^G93A^: 0.588 ± 0.5% adjusted *p*-value = 0.758, and PFN1^G118V^: 1.49 ± 1%, adjusted *p-value* = 0.868 Figure 3L). It was not possible to know the identity of these non-CSMN cells with utmost certainty because immuno-coupled EM allowed for one colorimetric reaction. However, since many different non-CSMN cells were analyzed (*n* = 51) and they all displayed very similar and comparable results, we feel confident to report that the stages of mitoautophagy is restricted to the mitochondria of diseased CSMN, and is excluded from a broad range of non-CSMN cells.

### Mitochondria in CSMN of hSOD1^G93A^ Mice Display Unique Abnormalities

**Figure 4:**
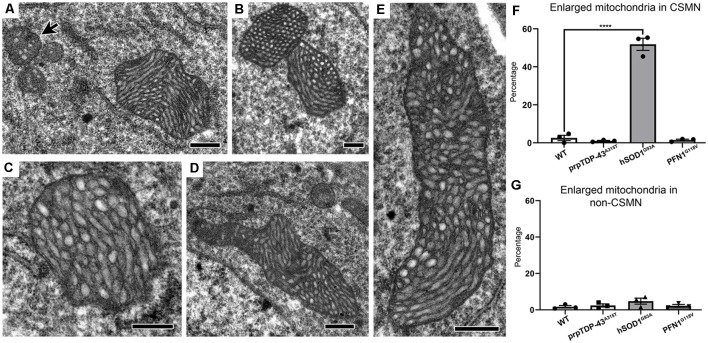
Mitochondria in CSMN of hSOD1^G93A^ mice are extensively enlarged at P15. **(A–E)** Representative images of enlarged mitochondria in CSMN of hSOD1^G93A^ mice. Arrow marks examples of normal size mitochondria. **(F)** Quantification of percentage of CSMN that contain enlarged mitochondria in WT, prpTDP-43^A315T^, hSOD1^G93A^ and PFN1^G118V^ mice. **(G)** Quantification of percentage of non-CSMN cells that contain enlarged mitochondria in WT, prpTDP-43^A315T^, hSOD1^G93A^ and PFN1^G118V^ mice. *p*-value **** <0.0001. Scale bar: **(A–E)** = 500 nm.

Not all mitochondrial abnormalities were shared or common among diseased CSMN. For example, mitochondria in the CSMN of hSOD1^G93A^ mice displayed a unique morphology. Many were extensively enlarged (Figures 4A–E), reaching about 20–30 times the size of normal mitochondria (Figure 4A, arrow). The cristae and the matrix structures were abnormal, but the inner membrane was intact. These mitochondria were not undergoing the stages of self-destruction as observed in CSMN of prpTDP-43^A315T^ mice. Of note, the enlarged mitochondria were present mostly in CSMN of hSOD1^G93A^ mice (WT: 2.57 ± 1%; prpTDP-43^A315T^: 1 ± 1%, adjusted *p*-value = 0.918; hSOD1^G93A^: 51.86 ± 3.2%, adjusted *p*-value = 0.0001, PFN1^G118V^: 1.46 ± 1%, adjusted *p*-value = 0.968; Figure 4F). Non-CSMN cells did not have these enlarged mitochondria (WT: 1.73 ± 1%; prpTDP-43^A315T^: 2.57 ± 1%, adjusted *p*-value = 0.954; hSOD1^G93A^: 4.09 ± 1%, adjusted *p*-value = 0.237 and PFN1^G118V^: 4.17 ± 1%, adjusted *p*-value = 0.980; Figure 4G).

**Figure 5:**
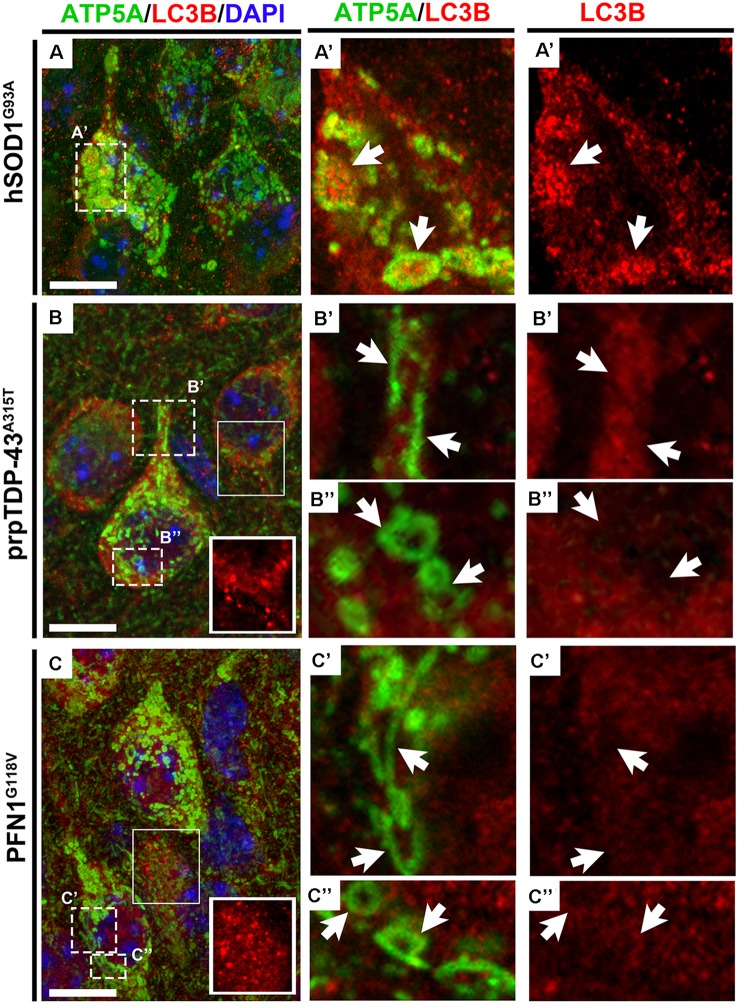
Mitochondria that undergo mitoautophagy lack direct interaction with autophagosomes. ATP5A expression profile recapitulates electron microscopy findings of mitochondrial defects in CSMN of hSOD1^G93A^ **(A)**, prpTDP-43^A315T^**(B)** and PFN1^G118V^ mice **(C)** in representative 100X images. LC3B expression marks the presence of autophagosomes in cells and ATP5A shows the location and the size of the mitochondria in CSMN. Enlarged mitochondria observed in CSMN of hSOD1^G93A^ mice are co-localized with autophagosomes (**A’**, arrows). However, mitochondria that undergo mitoautophagy in prpTDP-43^A315T^ mice (Stage I: **B’**, arrows; Stage II: **B”**, arrows) and PFN1^G118V^ mice (Stage I: **C’**, arrows; Stage II: **C”**, arrows) are not co-localized with autophagosomes, albeit autophagosomes in other nearby neurons are present (boxed areas enlarged within figure panel of **B**,**C**). Scale bar = 10 μm.

Since the size of the mitochondria was so extensively enlarged in the CSMN of hSOD1^G93A^ mice and they displayed a very distinct morphology in CSMN of prpTDP-43^A315T^ and PFN1^G118V^ mice, we were intrigued to visualize their interaction with autophagosomes using high magnification fluorescent imaging. Mitochondria were marked with the presence of ATP5A expression (Figure 5). LC3B expression was used to investigate whether mitochondria that undergo mitoautophagy are associated with LC3B+ autophagosomes. Mitochondria were detected in CSMN of hSOD1^G93A^(Figure 5A), prpTDP-43^A315T^(Figure 5B), and PFN1^G118V^ (Figure 5C) mice. Similar to observations made with EM, mitochondria in the CSMN of prpTDP-43^A315T^ and PFN1^G118V^ mice included mitochondria at Stage I (Figures 5B’,C’) and II (Figures 5B”,C”) of mitoautophagy. However, none of them (*n* = 21) had direct contacts with autophagosomes albeit nearby cells contained LC3B+ autophagosomes (boxed area enlarged within Figures 5B,C). This was in striking contrast to mitochondria of CSMN in hSOD1^G93A^ mice, which were found to be co-localized with LC3B+ autophagosomes (Figure 5A’).

Our detailed ultrastructural surveillance of mitochondrion in CSMN that become diseased due to different underlying causes in ALS at P15, reveal that mitochondrial defects occur much earlier than neuronal degeneration, and thus potentially contribute to upper motor neuron vulnerability. Interestingly, not all mitochondria undergo a similar mode of degeneration in all diseased upper motor neurons. Presence of mitoautophagy, a unique self-destructive path for mitochondria to eliminate themselves, is revealed mainly in CSMN of prpTDP-43^A315T^ and PFN1^G118V^ mice, and differs from the previously reported mitophagy.

## Discussion

Upper motor neuron degeneration is central to many diseases, such as ALS, HSP and PLS. Therefore, investigating the underlying causes of upper motor neuron vulnerability would have broad implications and is of great importance. There is growing body of evidence suggesting that energy metabolism contributes to ALS pathology when disturbed (Kasarskis et al., 1996; Vaisman et al., 2009; Granatiero and Manfredi, 2019), and has been compromised in ALS patients (Dupuis et al., 2011), who show severe functional decline and rapid deterioration in survival with a higher metabolic index (Steyn et al., 2018). Similar imbalances in energy homeostasis have been reported in mSOD1, and mouse models of TDP-43 pathology (Dupuis et al., 2004; Browne et al., 2006; Fergani et al., 2007; Chiang et al., 2010; Cistaro et al., 2014). The disturbed glucose metabolism leads to severe depletion of ATP in the central nervous system of ALS (Dalakas et al., 1987; Browne et al., 2006; Palamiuc et al., 2015). Additionally, hyperlipidemia also contributes to an energy imbalance in ALS patients (Lacomblez et al., 2002; Dupuis et al., 2008). Since mitochondria are at the heart of ATP production and lipid homeostasis, investigation of the health and function of this important organelle with respect to neuronal degeneration has been an important quest.

Mitochondrion is a dynamic organelle, which undergoes fission and fusion as well as morphological changes due to cellular stress, and these are related to its functional activities (Picard et al., 2013). In addition to ATP generation, mitochondria modulate the initiation of immune response *via* production of a variety of signaling molecules and metabolic reprogramming (Mehta et al., 2017; Singer and Chandel, 2019). Functional and structural abnormalities in mitochondria lead to decreased ATP production (Mattiazzi et al., 2002; Wiedemann et al., 2002) and a broad spectrum of functional impairments such as alterations in glucose metabolism, lipid and calcium homeostasis, induction of apoptosis, and production of reactive oxygen species (Duchen and Szabadkai, 2010).

Since mitochondria are compartmentalized and their function depends on the integrity of each compartment, the morphology of mitochondria is considered to be informative about the energy state as well as the extent and the type of intracellular defects (Hackenbrock, 1966; McCarron et al., 2013). For example, their morphological alterations affect energy production (Benard et al., 2007), permeability and leakage of mtDNA and other signaling molecules inside the matrix (Dimmer and Scorrano, 2006), initiation of apoptosis (Wang and Youle, 2009), innate immunity (Mehta et al., 2017), and generation of reactive oxygen species (Yu et al., 2006). Therefore, the shape and structural integrity of mitochondria have been closely monitored in many different cells and neurons in an effort to assess the rate and the extent of mitochondrial turnover, cellular stress and the stage of cellular degeneration (Ono et al., 2001).

Various defects were previously reported in the mitochondria of ALS patients’ spinal motor neurons (Sasaki and Iwata, 2007). Increase in quantity and profound swelling were observed especially in the presynaptic nerve terminals of patients (Siklós et al., 1996). However, the health and integrity of mitochondria in Betz cells of ALS patients have not been investigated in detail. Emerging evidence—especially within the context of TDP-43 pathology—began to reveal the presence of ultrastructural defects mainly at the site of inner membrane (Gautam et al., 2019). Similarly, CSMN of prpTDP-43^A315T^ mice, which recapitulates many of the clinical symptoms of ALS and ALS/FTLD patients with progressive upper motor neuron loss and motor function defects (Wegorzewska et al., 2009; Gautam et al., 2019), contain mitochondria which disintegrate and lose the integrity of their inner membrane—a pathology that is commonly observed in both Betz cells of patients and CSMN of mice (Genc et al., 2017; Gautam et al., 2019).

CSMN display massive mitochondrial defects especially towards the end-stage of the disease (Gautam et al., 2016, 2019). Therefore, it is not possible to reveal whether mitochondrial problems contribute to neuronal vulnerability or if they are the outcome of other ongoing problems. We thus reasoned that if intracellular and ultrastructural defects are detected at P15, these would precede many of the cellular problems, and may, in fact, allow the distinction between cause and effect.

We find major ultrastructural defects with mitochondria only in diseased CSMN. Other non-CSMN cells have healthy mitochondria, similar to that of WT CSMN. In contrast, diseased CSMN contain many mitochondria that are structurally defective, and most CSMN are caught in action as they undergo *mitoautophagy*, a novel path for systemic self-destruction. In Stage I, the mitochondria are elongated, and in Stage II they start to curl around themselves, eventually touching and combining the two ends, generating a ring-like structure at the center. When this structure is formed, mitochondria begin to disintegrate; first the inner membrane and the cristae are broken down and finally, the outer membrane is consumed, which marks Stage III. *Mitoautophagy* is different from mitophagy because rather than fusing with a lysosome or being engulfed by an autophagosome, mitochondria disintegrate from within. This mode of self-destruction does not require contact with a lysosome or the formation of an autophagasome since this has never been previously reported, we named the phenomena “*mitoautophagy*” to emphasize the fact that this is a self-destructive path and the mitochondria consume themselves from within *via* distinct stages.

Defective mitochondria are usually removed from the cells mainly by a well-orchestrated path of mitophagy, which when impaired leads to motor neuron degeneration in ALS (Edens et al., 2016; Granatiero and Manfredi, 2019). Many of the proteins that are actively involved in mitophagy, contribute to ALS pathology when mutated (Ramesh and Pandey, 2017; Nguyen et al., 2019). Stimulation of autophagy rescues motor function in a mouse model of ALS (Marrone et al., 2019). Thus, clearance of defective mitochondria, as well as other organelles and protein aggregates, appear to be a mechanism by which neurons and cells utilize to maintain homeostasis.

Mitochondria are known to undergo physiological changes as a response to stress-induced pathways. In the event of a reduction in membrane potential or disturbance in the electron transport chain, mitochondria can elongate in a process called hyperfusion to protect themselves against degradation and to stimulate ATP production (Rolland et al., 2013; Friedman and Nunnari, 2014). Enlarged mitochondria have been previously reported in the event of cerebral hypoxia-ischemia (Puka-Sundvall et al., 2000), and in the myocardium of aging and endurance-trained mice (Coleman et al., 1987). Similarly, mitochondria in CSMN of hSOD1^G93A^ mice were swollen and extensively enlarged at P15. Within the cell, the stress of energy deficiency leads to the phosphorylation and inactivation of DRP1, a pro-fission protein, leading to uninhibited mitochondrial fusion and generation of massively enlarged mitochondria, which can compact more cristae per unit area and thus increase the amount of ATP production (Gomes et al., 2011). In their enlarged state, mitochondria can also in part protect themselves from autophagy (Rolland et al., 2013) because autophagosomes at times fail to engulf such enlarged mitochondrion (Rambold et al., 2011). At P15, CSMN of hSOD1^G93A^ mice could be struggling to maintain cellular homeostasis and thus increase the size of their mitochondria to generate more energy and to avoid autophagy.

In striking contrast to the mitochondrial defects observed in CSMN of hSOD1^G93A^ mice, the CSMN of prpTDP43^A315T^ mice contain numerous mitochondria that are at different stages of mitoautophagy. The association of TDP-43 with mitochondria has been previously shown (Davis et al., 2018). Even though TDP-43 mainly resides in the nucleus, when it is in the cytoplasm it is located mainly in the mitochondria (Wang et al., 2013, 2016), more specifically in the matrix and the intermembrane space. Similar to mitochondrial defects, TDP-43 pathology is also a common denominator in many neurodegenerative diseases. There are many suggestive findings that these two could be linked or could develop simultaneously. For example, mitochondrial aggregates were detected in transgenic mice overexpressing WT form of TDP-43 (Xu et al., 2010), numerous mitochondrial defects are present in transgenic mouse models of TDP-43 as well as Betz cells of ALS and ALS/FTLD patients with TDP-43 pathology (Wang et al., 2013; Gautam et al., 2019). WT TDP-43 interacts with many mitochondrial proteins, such as PHB2 and VDAC1 (Davis et al., 2018), which are important proteins for the initiation of mitophagy. It is possible that mutant TDP-43 cannot properly interact with its binding partners in the mitochondria, which ultimately impact their structure and health. For instance, mutant TDP-43 interaction with Mfn2 is reported to induce mitochondrial fragmentation (Davis et al., 2018). Studies in neuron-like cell lines, mutant TDP-43 inside the mitochondria was also shown to bind to mitochondria transcribed mRNA that encodes the subunits of the oxidative phosphorylation system and impaired their assembly and thus function (Salvatori et al., 2018; Huntley et al., 2019). Mutant TDP-43 is reported to disrupt the mitochondria-ER interactions, which are important sites of lipid transfer between the two organelles (Stoica et al., 2014). Therefore, there are many possible ways that mutant TDP-43 impacts the stability, integrity and the health of mitochondria. In fact, a recent study has shown a direct link between the expression of mutant form of TDP-43 and the integrity of mitochondria in fly models. Increased production of ROS and activation of mitochondrial protein folding response were found to be involved in the observed defects (Wang et al., 2019). Here, we show that especially in the CSMN of prpTDP-43^A315T^ mice, there are major mitochondrial defects that occur very early in life and are restricted to CSMN at this age. Similar defects are not observed in the hSOD1^G93A^ mice, which are known to lack TDP-43 pathology, as in the case of ALS patients with SOD1 mutations (Mackenzie et al., 2007; Robertson et al., 2007).

Mutations in the *Profilin 1* gene have been detected in ALS patients (Wu et al., 2012) and mouse models are developed to investigate the cellular and molecular basis of neurodegeneration. Because profilin is an actin-binding protein, this model is built to recapitulate disease pathology that develops mainly due to cyto-architectural defects (Fil et al., 2017). CSMN undergoes progressive degeneration in the PFN1^G118V^ mice, albeit at later ages (Fil et al., 2017). Mitochondrial defects have not been previously reported to be one of the contributing causes of motor neuron degeneration with respect to lack of profilin function. However, defects in actin cytoskeleton contribute to localization and mis-localization of organelles inside the cells, affecting their function. Therefore, investigation of mitochondria in PFN1^G118V^ mice was of interest. Even though mitochondria undergoing mitoautophagy were detected, the mitochondrial defects were not pronounced in the CSMN of PFN1^G118V^ mice at P15, and this could be due to the fact that CSMN degeneration occurs later in life in the PFN1^G118V^ mice (i.e., P135; Fil et al., 2017). However, it is important to note that the mode of mitochondrial degeneration in CSMN of PFN1^G118V^ mice showed similarities to that of prpTDP43^A315T^, but not to that of hSOD1^G93A^ mice. Understanding the basis of profilin mutation-mediated motor neuron degeneration is an emerging field and it appears to share common biology with TDP-43 pathology. TDP-43 pathology is observed in the brains of patients with PFN1 mutations (Tanaka et al., 2016), and mutant PFN1 induced accumulation of TDP-43 and most interestingly facilitated conversion of WT TDP-43 into an abnormal form, which formed aggregates. Therefore, the association between TDP-43 and profilin is of interest. Here, we find that the mitochondria in the CSMN of PFN1^G118V^ mice display more resemblance to that of prpTDP43^A315T^ mice, as they both have mitochondria that undergo mitoauthophagy at a very early age. One possible explanation could be that in the presence of PFN1 mutation, the WT TDP-43 may well be converted into its mutant abnormal form in CSMN, as has been reported in other cells (Tanaka et al., 2016), and this may lead to the development of similar mitochondrial defects observed with respect to TDP-43 pathology. Further detailed cellular analyses are required to reveal the association between profilin and TDP-43 pathology.

Our results reveal the presence of mitochondrial defects as early as P15 in CSMN and the contribution of mitochondrial dysfunction to upper motor neuron vulnerability, especially within the context of TDP-43 pathology. We also report *mitoautophagy*, a unique self-destructive path that leads to disruption of mitochondria, without a need to contact with an autophagosome. Even though mitochondria of CSMN in hSOD1^G93A^ mice are extensively enlarged, they display profound defects in prpTDP43^A315T^ mice. The presence of subtle, but significant differences between mitochondrial defects observed in upper motor neurons, also set the stage for our understanding of the common and unique mechanisms responsible for their vulnerability. This is critically important for identifying novel targets for drug discovery efforts and for building effective treatment strategies for diseases in which voluntary movement is affected.

## Data Availability Statement

The datasets generated for this study are available on request to the corresponding author.

## Ethics Statement

The animal study was reviewed and approved by Northwestern University Animal Care and Use committee.

## Author Contributions

PO and MG designed the study. MG, NK, and EX performed the experiments. PO, MG, NK, and EX analyzed the data and wrote the manuscript.

## Conflict of Interest

The authors declare that the research was conducted in the absence of any commercial or financial relationships that could be construed as a potential conflict of interest.
