# Supplementary material for: Mitoautophagy: A Unique Self-Destructive Path Mitochondria of Upper Motor Neurons With TDP-43 Pathology Take, Very Early in ALS
Source: Front Cell Neurosci. 2019 Nov 7;13:489. doi: 10.3389/fncel.2019.00489 (PMC6854036; doi:10.3389/fncel.2019.00489)
Supplement: Supplementary file 1 [file Table_1.DOCX]

Supplementary Table 1

| **Genotype** | **WT** | **prpTDP-43^A315T^** | **hSOD1^G93A^** | **PFN^G118V^** |
| --- | --- | --- | --- | --- |
| Number of mice | 3 | 3 | 3 | 3 |
| Total number of cells counted | 56 | 62 | 50 | 58 |
| Total number of mitochondria counted | 1440 | 1611 | 827 | 1146 |
